# In Search of a Good Person-to-Job Fit: Perspectives of Autistic Clients, Their Family Members, and Employers of a Pilot Autism-Specific Employment Program

**DOI:** 10.1089/aut.2023.0075

**Published:** 2025-10-24

**Authors:** Jill Ashburner, Emily Glastonbury, Natasha Bobir, Davina Sanders, Elena Swift, Emily Rietberg, Jessica Hill

**Affiliations:** ^1^ Research and Development Department, Autism Queensland Limited, Sunnybank, Australia.; ^2^ The School of Health and Rehabilitation Sciences, The University of Queensland, St Lucia, Australia.

**Keywords:** autism, employment, inclusion, disability, employment service provider, workplace

## Abstract

Employment is an important factor contributing to the quality of life of autistic individuals. However, autistic people are substantially underrepresented in the workforce, and are more likely than non-autistic people to be in part-time/casual positions and/or in jobs for which they were overqualified. Current employment programs often cater to a limited range of autistic jobseekers. This project explored participants’ perceptions of an autism-specific, client-led employment program, Autism EmployABLE, with respect to (1) the program’s helpfulness and aspects that could be improved and (2) the clients’ happiness, confidence, and feelings of independence after gaining employment. We employed a hybrid approach for content analysis of semi-structured interviews to explore the perspectives of 15 autistic clients, 6 family members, and 5 employers. Interview data revealed that almost all participants perceived *Autism EmployABLE* to be helpful, and that it aligned well with the Person–Environment–Occupation (PEO) Model of Occupational Performance, in that it addressed the interaction between person (e.g., interests, strengths), environment (workplace factors), and occupation (e.g., job tasks). Perceived benefits of the program included enhanced client confidence and independence, and improved outcomes compared with generic disability employment services. Suggested improvements included opportunities for clients to meet and share experiences, access to industry-specific mentoring, provision of written materials, and aptitude testing for specific roles. This study provides preliminary support for the benefits of *Autism EmployABLE* and highlights the importance of sourcing positions that match each client’s skills and interests to achieve the outcome considered critical to employment success—good person-to-job fit.

**Community Brief:**

**Why was this program developed?**

Despite the many strengths that autistic people can bring to the workplace, they are more likely than non-autistic people to be unemployed or to work in part-time/casual positions and/or in jobs for which they were overqualified. We developed *Autism EmployABLE* to help autistic people find satisfying and sustainable employment that matches their skills and interests.

**What does the program do?**

The program aims to support autistic jobseekers to find satisfying jobs by matching their interests, strengths, and support needs (the person) with characteristics of the workplace (the environment) and job tasks (the occupation). The program is staffed by professionals with autism-specific knowledge (e.g., psychologists, occupational therapists, teachers) and staff with experience in the vocational sector and marketing skills. The staff therefore have the expertise to work effectively with both clients and businesses. Clients take a leading role by setting their own employment goals and developing “roadmaps” to help them communicate about their strengths and support needs. The program supports clients to apply for open employment opportunities that suit their skills and interests.

**How did the researchers evaluate the new program?**

We asked the autistic clients who accessed *Autism EmployABLE*, their family members, and employers to participate in an interview, which explored the helpfulness of the program in finding a job and navigating the workplace, satisfaction with job placements, and client well-being after finding employment. We asked about aspects of the program that they liked and aspects that could be improved.

**What were the early findings?**

The findings suggest that a good person-to-job fit can be achieved by matching the skills, interests, and support needs of clients with the needs of businesses. Participants observed improvements in the clients’ confidence and independence. They reported achievement of better employment outcomes than they had achieved through generic disability employment services. Suggested improvements included more opportunities for clients to meet and share experiences. Clients seeking employment in professions such as engineering or information technology (IT) would like access to mentors with profession-specific knowledge. One employer suggested aptitude testing for specific roles.

**What were the weaknesses of this project?**

We are unsure whether the perspectives of the program reported here can be generalized to other cultures and communities.

**What are the next steps?**

This study supports autism-specific, client-led employment programs that consider the unique skills and interests of autistic clients when finding a suitable job. Many of the clients and their family members perceived that the clients achieved more satisfying and sustainable employment through this program than through generic disability employment services. The next step would therefore be to provide more autistic jobseekers with access to this type of program.

**How will this work help autistic adults now or in the future?**

If autistic people are supported to find employment that matches their skills and interests, they are more likely to find satisfying jobs and are more likely to be happy to continue working in these positions.

## Introduction

Employment is a significant concern within the autism community, as autistic people are substantially underrepresented in the workforce (the term “autistic” reflects the preference of many autistic adults for identity-first language^1^) In a study of the UK autism community’s views on autism research, both autistic adults and parents of autistic children identified employment as a priority.^2^ The Australian Bureau of Statistics (ABS) Labour Force Framework^3^ classifies the working age population (aged 15–64) into three mutually exclusive categories: *Employed* (working ≥1 hour per week), *Unemployed* (not currently employed but available for and looking for work), and *Not in the Labor Force* (people not currently looking for work such as students or carers). In 2018, the ABS^4^ reported a labor force participation rate of 38.0% among autistic people, compared with 53.4% of people with disabilities in general and 84.1% of people without disability, while the unemployment rate for autistic people was 34.1%, compared with 10.3% of people with disabilities in general and 4.6% of people without disability. Low employment levels among autistic people have also been reported internationally. A nationwide US study involving 3520 autistic adults without an intellectual disability reported a 23% employment rate,^5^ while a 24% employment rate was reported in 154 Japanese autistic adults.^6^ With respect to Australian autistic people without intellectual disability, one survey reported that 54% of 267 respondents not in full-time education had paid employment,^7^ while another reported that 48% of 95 respondents had paid employment.^8^ High rates of underemployment are also reported in this population. Baldwin et al.^9^ found 45% of 249 Australian autistic adults without intellectual disability were overrepresented in part-time and casual positions and/or in jobs for which they were overqualified. Likewise, Harvery et al.^10^ reported that autistic adults were more likely to work part-time, reduced hours, and/or at skill levels lower than their qualifications warranted.

### Strengths and challenges of autistic employees

Autistic people are reported to have strengths that are valued in the workplace.^11^ A survey of the perceptions of Australian employers indicated that autistic employees demonstrated better attention-to-detail, work ethics, and quality of work than non-autistic employees.^12^ Similarly, a survey of 66 autistic employees revealed self-perceptions of employment-related strengths, including creativity, focus, memory, honesty, and dedication.^31^ Bury et al.,^14^ however, caution against blanket assumptions about strengths that may not apply to all autistic people and advocate an individualized approach for matching strengths to job roles.

With respect to challenges, a qualitative exploration of the perspectives of 29 autistic employees and 15 supervisors indicated that some workplaces do not effectively accommodate the social-communication differences of autistic employees (e.g., unnecessary expectations to participate in workplace social events).^15^ Similarly, a survey by Neary et al.^8^ revealed that those who found employment often worked in jobs for which they were overqualified, because workplaces allowing for higher level employment did not cater to their social-communication differences. Bury et al.^15^ observed that workplace social challenges can result in negative self-attributions by autistic employees, and therefore hypothesize that these challenges may negatively impact mental health.

Notwithstanding these challenges, enhanced participation in employment has been associated with improvements in emotional well-being. Participants of a qualitative study by Hedley et al.^16^ perceived improvements in sense of purpose, personal independence, and social relationships in autistic trainees who participated in a 3-year employment and training program. Mason et al.^17^ found employment to be a significant positive predictor of the quality of life of autistic people. Baldwin et al.^9^ observed that many employed autistic people perceived work as providing an opportunity for self-actualization through which they could apply their knowledge, skills, and interests.

### Factors that contribute to successful employment

Dreaver et al.^18^ explored factors that contribute to successful employment of autistic people from the perspective of Swedish and Australian company directors and who had employed an autistic person. Factors perceived as critical to success included the following: (a) autism awareness programs to ensure that coworkers have a sound understanding of autism; (b) optimization of the work environment through the provision of regular feedback, clear instructions, structure and predictability, and reductions in distracting sensory input; and (c) a good person-to-job fit. Likewise, an international survey of 687 participants, including autistic individuals, family members, employers, and service providers, reported that a good person-to-job fit and a focus on strengths were perceived to be important.^19^ In contrast, they perceived stigma, a lack of understanding of autism, and communication challenges to be barriers. A qualitative exploration of the perspectives of 19 autistic employees reiterated the importance of a good person-to-job fit, in addition to a workplace climate of acceptance and respect.^20^ Similar to Dreaver et al.,^18^ Waisman-Nitzan et al.^20^ described the need for workplaces to offer clear instruction and feedback, structure, and reductions in distracting sensory input.

### Employment programs for autistic people

Several international studies report on autism-specific employment services. A UK study by Mawhood and Howlin^21^ compared the employment outcomes of 16 autistic individuals who participated in an autism-specific employment program, with a control group of 20 autistic individuals with similar intellectual and linguistic abilities and employment history. The autism-specific program entailed support worker assistance with job finding, work preparation, 2–4 weeks of job coaching, and education of employers and coworkers. The participants in the autism-specific employment program achieved job placement outcomes (63%) that were superior to those of the control group (25%).^21^ The autism-specific program also proved to be more successful in securing job placements that were commensurate with the clients’ intellectual and educational levels, resulting in higher salaries and job satisfaction. Thirteen businesses (mostly multinationals) engaged with the program and provided job opportunities (predominantly administrative, clerical, and IT positions).

Some autism-specific employment programs focus exclusively on young autistic people after leaving school. For example, Wehman et al. completed a randomized control trial of a US employment program for autistic people aged 18–21 years.^22^ The program involved business-led intensive internships in specific industries (hospitals, bank centers, or government centers). Eighty-5% of intervention group participants subsequently acquired employment compared with 6% of the controls.

Specialisterne, an organization that perceives the talents of autistic people (such as attention to detail) as a competitive advantage, has influenced recent employment programs.^11^ Three Australian studies reported on businesses that have collaborated with Specialisterne Australia on recruitment, selection, and training of autistic job candidates.^16,23,24^ Both Hedley at al.^16^ and Flower et al.^23^ reported on programs that offered a limited number of positions and that were therefore competitive. Hedley et al.^16^ explored the perspectives of autistic employees working in a software test analysis business. Of the 63 original applicants, 14 participated in a 4-week assessment and training program, 11 of whom were offered positions. A follow-up study reported a strong improvement in employability with the unemployment rate decreasing to 16% following the program for all participants (including participants who exited the training before completion).^25^ Flower et al.^23^ reported on a business that aimed to employ autistic people as records management officers. Of 52 original applicants, 12 attended a 3-week training assessment, 8 of whom were offered positions. Similarly, Gatfield and Falkmer^24^ reported on a program that employed autistic people to work in animal care (piggeries). The autistic employees, family members, and mentors/supervisors/managers involved with this program perceived the modified recruitment process, orientation and training weeks, workplace mentors, and positive work environment to be helpful. In recognition of the challenges that interviews can create for autistic clients, Specialisterne runs 3- to 4-week assessment and training programs with the intention of providing a preview of the role, as an alternative to interviews. However, some autistic clients perceive these training and assessment programs to be stressful: *like interview stress but spread over 3 weeks*.^23^

In Australia, autistic people frequently access government-funded generic employment services for people with disabilities, called Disability Employment Services (DES).^27^ These services offer assistance in preparing for, finding, and keeping a job to people with disabilities, injuries, or health conditions. In most cases, they are not staffed by professionals with expertise in autism. Hayward et al.^28^ found that autistic DES users rated the DES providers as low on (a) their ability to work with autistic people, (b) their understanding of autistic people’s work needs, and (c) their autism knowledge. They also raised concerns that DES providers may incentivize job placement over job-fit to receive funding. To achieve sustainable employment outcomes for autistic clients, Hayward et al.^28^ recommend vocational services that specialize in a disability type (such as autism), that DES staff receive more autism-specific training and support from a reputable source, and the use of a nontime-restricted, customized employment approach to achieve sustainable employment.^28^

### Emerging practice: Autism EmployABLE

This article reports on participants’ perceptions of an emerging practice, *Autism EmployABLE*, during its first year of operation in 2020. The program continues to operate on a full-time basis in 2024. Australian Government grants have paid for the Autism EmployABLE staff and operating costs. Autism EmployABLE supports clients to find open employment based on their skills and qualifications. Successful jobseekers are paid by their employer. One of the 15 clients who participated in this study received a government wage subsidy for 26 weeks to support onboarding and training.

The team leader and staff worked collaboratively to develop Autism EmployABLE to address the need for an employment service that achieves a good person-to-job fit by addressing the interaction between the person (e.g., interests, strengths), environment (workplace factors), and occupation (e.g., job tasks). Two novel aspects of the program that facilitated this process include (a) a staffing mix that includes professional staff and staff with experience in the vocational sector and (b) a client-led, person-centered focus.

The staffing mix of Autism EmplyABLE includes professionals with autism-specific knowledge (e.g., psychologists, occupational therapists, and teachers) and staff with experience in the vocational sector and marketing skills. The program is embedded within an autism service provider with the capacity to provide extensive autism-specific staff training. The staff therefore have the autism-specific expertise to work collaboratively with clients to gain a detailed understanding of their skills, interests, and support needs, in addition to the skills to work with businesses (e.g., to approach businesses, gather information on business needs, provide autism awareness training for businesses, and recommend autism-specific workplace accommodations). This understanding of the needs of both clients and businesses is used to support clients in finding open employment opportunities that match their interests, skills, and qualifications. A further advantage of being embedded within an autism service provider is that clients who require additional support before accessing Autism EmployABLE can be referred to other services to develop foundational employment, independent living, or communication skills.

The second novel aspect of the program is that it is client-led and person-centered by which we mean that the employment support process begins with the client. The client is encouraged to take a leading role, working collaboratively with staff to develop a shared understanding of their aspirations, strengths, interests, and support needs, with the aim of sourcing job opportunities to suit their unique requirements. There is an emphasis on self-determination and equipping clients with the self-advocacy skills that they will require throughout their working lives. This approach differs from the employment programs reviewed above,^16,21–24^ which are largely business-led in that they first identify a business with various roles to offer, before seeking autistic clients who suit these roles. Although business-led employment programs can offer a range of roles, they often engage with specific businesses that offer IT and administrative jobs. However, these roles may not suit many autistic jobseekers, such as those who experience literacy challenges or who prefer working outdoors. In contrast, as Autism EmployABLE is client-led, there is greater emphasis on client’s needs and preferences. Baldwin et al.^9^ found that autistic people engage in a wide range of professions that often do not conform to “stereotypical” employment categories associated with autism (such as IT). Bury et al.^26^ argue that if employment programs focus exclusively on a narrow range of jobs that are assumed to suit autistic people, they risk failing to accommodate the diverse interests of autistic people.

Autism EmployABLE is based on the Person–Environment–Occupation (PEO) Model of Occupational Performance,^29^ which proposes that improved compatibility between three components (person, environment, and occupation) leads to improved occupational performance. The application of the PEO model to Autism EmployABLE was inspired by the occupational therapists involved in the program’s development, as this model is central to the clinical reasoning of occupational therapists. When applied to employment, a higher level of congruence between the person (vocational interests and strengths, personality, and competencies), the environment (social, attitudinal, cultural, and physical attributes of the workplace), and the occupation (job role, activities, and tasks) is likely to enhance person-to-job fit, which in turn leads to enhanced occupational performance.

The PEO model has underpinned two previous studies focusing on employment of autistic people.^30,31^ Waisman-Nitzan et al.^30^ used the PEO model to explore the work-related challenges faced by 47 Israeli autistic employees without cognitive disabilities. Each participant completed self-reported assessments of personal, occupational, and work environment characteristics, as well as work performance and self-efficacy assessments. The authors found significant correlations between each PEO dimension, and work performance and self-efficacy. A scoping review framed facilitators and barriers to employment of autistic adults using a PEO model.^31^ For example, person-related facilitators may include job-specific skills, while barriers may include challenges in coping with unexpected workplace changes. Examples of work-related (occupational) facilitators include support with job search and interview preparation, whereas barriers include unclear expectations and schedules. Examples of environmental facilitators include compatible sensory environments, while barriers include negative coworker attitudes. In accordance with the PEO model, Autism EmployABLE focuses on the interaction between the following:

- *The person (client):* Professional staff support the clients to identify and prioritize their own employment-related goals using a picture-based, card-sort tool called the *Adolescent Adult Goal Setting Tool.*^32^ They also support the clients to develop individual “roadmaps” that convey their preferences for communication, social interaction, executive functioning, emotion regulation, sensory and information processing style, and other job-specific factors (e.g., preference for office-based vs. outdoor environments). The clients complete these roadmaps over several sessions with input from professional staff based on their observations of client strengths and characteristics. The roadmaps function as an ongoing tool to facilitate communication with employers around job preferences and potential supports. The professional staff support the clients to explore their own neurodivergent traits, and to develop self-advocacy skills that help promote their marketable strengths as well as communicate areas in which they wish to receive more support. In addition, clients work on self-identified employment-related goals such as understanding workplace expectations. Where required, Autism EmployABLE sources vocational education externally that aligns with their employment-related goals.
- *The environment (workplace):* The staff approach the employers to participate in multiple ways, including face-to-face interactions, cold calling, direct marketing of the clients’ skills, and offers of free autism awareness sessions. Autism awareness sessions for employers focus on (a) their capacity to work effectively with autistic employees, (b) ways to address workplace social, attitudinal, and cultural barriers, and (c) the benefits of a neurodiverse workforce, reflecting neurodiversity within the general population. Autism EmployABLE staff also often find it necessary to correct erroneous assumptions of employers (e.g., assumptions that all autistic people have an intellectual disability, or that their skills and interests are only in IT-related fields). Autism EmployABLE staff highlight the strengths of autistic people and reframe their social-communication differences. Recent research suggests that non-autistic people have as much difficulty understanding autistic people as *vice versa* (labeled a double empathy problem).^33^ Workplace communication is therefore approached from a two-way perspective, by encouraging both autistic employees and their coworkers to accommodate one another’s communication preferences. For example, coworkers learn to provide clear, unambiguous instructions, while autistic employees learn about lunchroom expectations. Autism EmployABLE staff also gain an understanding of the business’ needs, to recommend suitable candidates.
- *The occupation (job role):* Autism EmployABLE staff support clients to search for and apply for suitable open employment opportunities that suit their unique strengths and interests, and to prepare for interviews. Where appropriate, Autism EmployABLE staff negotiate with businesses to create bespoke roles (e.g., part-time roles involving components of existing roles that align with the person’s strengths). The staff analyze job tasks to determine the need for accommodations (e.g., the provision of step-by-step instructions). Recommended accommodations are commonly beneficial to both autistic and non-autistic employees. The staff also provide job coaching when clients start work and continue as needed.

### Research questions

In this study, we aimed to explore the participants’ perceptions of Autism EmployABLE by addressing the following research questions:

1. How do Autism EmployABLE clients, their family members, and employers perceive Autism EmployABLE, in terms of its helpfulness and aspects that could be improved?
2. What perceptions did Autism EmployABLE clients and their family members have of the clients’ happiness, confidence, and feelings of independence after gaining employment?

## Evaluation Method

This qualitative evaluation gathered data from semi-structured interviews, as is considered appropriate when gathering the perspectives of participants in real-world settings.^34^ The University of Queensland Human Research Ethics Committee provided ethical approval. The research team used purposive sampling to recruit clients with a range of ages, co-occurring conditions, and educational levels, and employers from a range of industries. We used easy-read participant information sheets and a short video to explain the study to clients. We gave clients the option of consenting to the research team inviting a family member to participate in the research. All participants provided informed written consent. We stored all data securely so that these were accessible to only the researchers. Three authors (J.A., N.B., and D.S.) developed the interview questions for Autism EmployABLE clients, family members, and employers. We reviewed the questions after trialing them with one client and one family member, but no changes were required as they elicited sufficient information. See the interview questions in the Supplementary File S1. The second author (E.G.), who had no direct involvement with the program delivery, was employed to conduct semi-structured interviews via telephone or videoconferencing. E.G. is an experienced teacher with research officer experience in qualitative research. The following aspects of the methodology enhance credibility and trustworthiness:

• A member checking procedure involved one representative from each participant group reviewing the synthesized and analyzed data. Each representative agreed that the categories accurately represented their experiences.
• Data-source triangulation was established by gaining the perspectives of multiple stakeholder groups (clients, family members, and employers).
• Purposive sampling ensured that a diversity of participant voices was heard.
• Four researchers independently coded the data.
• An additional researcher provided peer debriefing.

### Participants

We interviewed 15 clients (14 males and 1 female). They all met the eligibility criteria for Autism EmployABLE (≥18 years of age, having a self-reported autism spectrum disorder diagnosis, and seeking ≥8 hours of open employment per week). The clients provided details on their autism diagnosis (e.g., autism spectrum disorder, Asperger’s disorder), age when diagnosed, and the diagnostician’s profession (e.g., pediatrician, psychiatrist, psychologist). Their ages ranged from 19 to 41 years (M = 25.47, SD = 6.62). Thirteen client participants had co-occurring conditions, including anxiety disorder (*n* = 7), attention deficit disorder (*n* = 5), obsessive compulsive disorder (*n* = 4), mood disorder (*n* = 2), intellectual disability (*n* = 1), and bipolar disorder (*n* = 1). Their highest level of education included secondary school completion (Queensland Certificate of Education) (*n* = 3), vocational education certificates (*n* = 3), tertiary diploma (*n* = 1), and bachelor’s degrees (*n* = 8). The reasons for these clients seeking support included lack of success with generic disability employment services, limited work history, lack of prior job suitability, failure of previous workplaces to accommodate needs, and workplace bullying. Outcomes achieved by these 15 clients through Autism EmployABLE included 13 securing some form of employment, one engaging in vocational training, and one engaging in volunteer work in his field. Of the 13 clients who secured employment, 10 gained ongoing paid employment and 3 gained short-term contracts. Two of the clients who gained employment were also engaged in training.

Six family members (five mothers and one father) who were in daily contact with the clients, consented to participate, with the permission of their children who were Autism EmployABLE clients. We also interviewed five employers of Autism EmployABLE clients. They came from a range of businesses, including digital signage, microscope supplier, homewares, roofing and cladding, and water quality analysis equipment.

Six Autism EmployABLE staff members also provided written informed consent to participate in semi-structured interviews and focus groups. Their perceptions are described in a separate article. The professional staff members included a team leader with a Bachelor of Science and Graduate Diploma of Psychology and 8 years of vocational services experience. Other professional staff included an occupational therapist with 2.5 years of experience; a teacher with a Master of Special Education and 20 years of experience; a teacher/developmental educator with a Masters of Autism Studies and 22 years of experience; and a school psychologist (Education Specialist in School Psychology, USA) with 15 years of experience. An employment engagement officer with 2 years of experience in vocational services was also employed primarily to approach potential employers.

### Data analysis

We transcribed the recorded interviews verbatim. We used a hybrid deductive and inductive approach to content analysis drawing on the work of Fereday and Muir-Cochrane^35^ and Hsieh and Shannon.^36^ This involved a balance of deductive coding derived from a theoretical framework (in this case, the PEO model to Autism EmployABLE), and inductive coding applied iteratively throughout the analysis. E.G., N.B., and J.A. manually coded 100% of the transcripts independently. The researchers worked together to establish a coding dictionary, which included clear definitions and descriptions of each code. We met on multiple occasions to review and refine the codes using the constant comparison method and discuss until consensus was met.^34^ We grouped the codes into categories based on the PEO model. Inductive coding was applied iteratively throughout the analysis allowing additional categories to emerge organically from the data. J.A. and E.S. rereviewed and discussed the final categories over several meetings, until consensus was achieved. E.R. provided independent peer debriefing. We judged that we had reached the point of data saturation as considerable repetition was evident in the data.^37^

## Results

Figure 1 provides an overview of the results, while Tables 1 to 3 detail illustrative quotes supporting the categories and subcategories. Deductive analysis involved coding of the data into the PEO model categories—Person, Environment, and Occupation (see Table 1 detailing illustrative quotes). Inductive analysis further revealed categories related to the EmployABLE outcomes (see Table 2) and satisfaction with Autism EmployABLE (see Table 3) that emerged from the data.

**FIG. 1.**
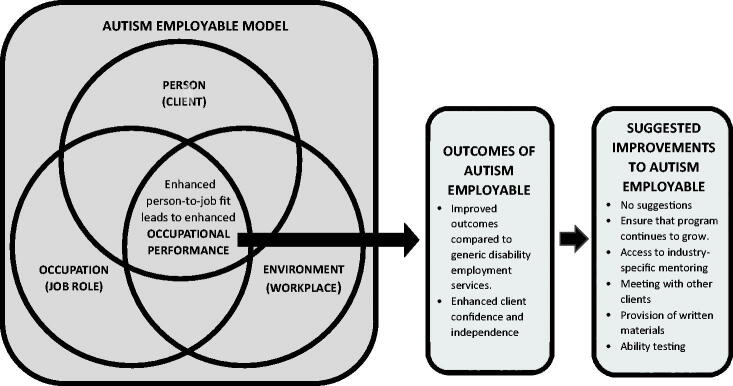
Application of PEO model of Autism EmployABLE, outcomes of Autism EmployABLE, and suggested improvements. PEO, Person–Environment–Occupation.

**Table 1.** Quotes from Clients (CL), Family Members (FM), and Employers (EM) that Illustrate PEO Model Used by Autism EmployABLE (AE)

Category	Subcategory	Example illustrative quotes
Person	**Understanding person and supporting self-understanding** (e.g., understanding strengths, interests, autism diagnosis)	**CL02:** *She* (AE staff member) *does understand autism and the quirks of autism and has patience with it.* **CL03:** *Just talking through my diagnosis… with different people has helped. I only got my diagnosis about 18-ish months ago. So, I know a bit more of the terminology.* **FM02:** *They spend more time with them, and working out their strengths, and focusing on that.* **FM04:** *They listen to what fears he had, what was going to work for him, what wasn’t going to work for him, what his interests were.* **EM01:** *It was more around… being…more aware of the needs of the individual candidates that we were evaluating to bring into the business.*
	**Client-led goals** (setting and working on goals)	**CL03:** *My personal goals… are getting my (bachelor) degree done, as well as moving out of my parents’ home. This program has helped me… realize some of the intermediate steps that I need to do in order… to gain employment first, to move out*. **CL05:** *They helped me with setting goals*. **FM04:** *It was giving* (client) *cards to put down on a table.* “*Let’s group them into what interests you, what’s important to you*.” **FM05:** *They’re teaching him to set goals, so that he felt like he was achieving stuff out here on his own, so that was really helpful.*
	**Developing skills** (foundational skills or externally sourced vocational training)	**CL03:** *I did do work specifically about disclosing my diagnosis. So that has helped me.* **CL04:** *She* (AE professional Practitioner) *has helped me with time management. That’s something I’m trying to work on*. **CL09:** *I did a Cert 3 in Warehousing and even got my forklift license - we met up in person to help with the final assessment for the license, because first time I failed and then I had asked for some help, and she was more than happy to help me out.* **FM01:** *It was tailored around* (client) *helping to learn about himself and what he might need in a job situation… navigating other aspects of the work, like what is appropriate socially*.
	**Supporting emotional well-being** (e.g., confidence, motivation)	**CL01:** *They gave me courage …for those job interviews and things like that*. **CL02:** *I was at my wit’s end before and there’s still no one else helping … without that help, I don’t know?* (AE professional practitioner) *was very helpful… I know she was a psychologist… golly gosh it was helpful.* **CL09:** *Thanks to* (AE professional practitioner)*, it’s really helped me up to get back on my feet and that’s made me feel a lot better …* **CL10:** *The support made me feel excited about working, even if I was a little nervous at the start.* **FM02:** *They’ve probably helped him get more motivated… Building up his self-confidence again and his self-worth and making him feel valued*.
	**Encouraging independence** (e.g., in seeking and acquiring jobs)	**CL08:** *With Autism EmployABLE’s;help, I’m confident that I’ll be able to make my way into the workforce.* **FM02:** *They’ve given him tools to look for work*. **FM04:** AE professional practitioner said, “*Let’s just let them go and have him (client) get the chance to just speak up and not everybody try to speak for him.”* **FM05:** *It was brilliant … helping him become more independent in his decision-making processes. EmployABLE gave him the space to do some of his own problem solving, but in a structured way*.
ENVIRONMENT	**Employers’ autism awareness** (overcoming attitudinal barriers, valuing diversity)	**CL03:** *There’s always that fear that once someone knows you’re on the autism spectrum, they just want to say “Yeah, okay. You’re defective”. But having someone who actually can advocate, that’s had some standing in the community say,” This is what autism employable people are good at”. They* (AE staff) *work with the employers to bring the staff up to speed.* ***C*L11:** *The more people you get in the program and the more connections you make … they’ll realize that people on the spectrum are valuable employees and can actually have really good skills***.** **FM02:** *They* (AE staff) *are working with this company to train them on working with people on the spectrum.* **EM03:** *Having someone with autism on the team, it does add that diversity.*
	**Supportive supervisors and coworkers**	**CL03:** *They seem very supportive. They come check on me every now and then, ask me if I’m having any problems.* **CL06:** *What I’m happy about was that with my supervisor, they will give me some feedback that I could use to improve my skills and later on in the weeks, they’re going to teach me more stuff*. **CL13:** *Very supportive actually, which has been really good. My managers are pretty understanding.*
	**Understanding the needs of businesses**	**EM01:** *The candidate who we placed; I think he was a very astute recommendation … his strengths line up very neatly to what we need for the business*. **EM02:** *She* (AE professional practitioner) *made a very quick assessment on what I needed... actually, I think she had a really good understanding of the strengths and weaknesses of the people she was working with.*
OCCUPATION	**Finding roles to suit person** (to suit interests, strengths skills, qualifications)	**CL01:** *I love to do that job* (groundsman) … *I don’t want to work behind a desk*. **CL09:** *She was the one that helped me with thinking that forklifting may be a good career choice for me, ‘cause we took a bit of a little career test … what I liked in certain categories* **CL13:** *The (*other*) job providers …they help you find work… mostly labour hire jobs... But Autism EmployABLE actually helps you find an employer… So, hiring for your skills, not for another pair of hands.* **FM04:** *The job is… a perfect fit for him. He obviously had listened to what* (client) *was after. We couldn’t be happier, and neither could our son.* **EM01:** *I don’t think I would have been able to find a better suitable candidate had I gone into I guess a more general or exhaustive search.*
	**Support in logistics of getting a job** (applications, interviews, job trials, starting work)	**CL02:** *Now I’ve got 10 different cover letters sitting here… I go and find the most appropriate one and then I alter it and it takes a few minutes but that ends up being a unique cover letter for every job, and it’s very helpful.* **CL04:** *They’ve just been giving me the* (interview) *question and asking me to respond to it and giving me advice on my answer.* **CL06:** *During the interview, they were present and taking notes down as I answered their interview questions. And once I’ve finished it, they’ll give me feedback on my interview on what areas I could improve on…to increase my chances of landing a job successfully.* **EM04:** *I think the best part of the experience was how prepared for the interview and for the work role the two candidates were before we actually engaged in recruitment. You could tell they’d been really briefed.*
	**Ongoing support**	**CL12:** *They (AE staff) do check up on me regularly, which is really good, to see how I’m doing. They’re not going to just abandon you after you’ve gotten a job.* **FM04:** *She’s still contacting him…. just to see how he’s going. He knows that if there’s any worries, he can call her.* **EM03**: *They continue to keep in touch with me as well and see how life is going.*
	**Workplace adjustments**	**CL03:** *If you really want to help, then you can give me concise orders that have been broken up into sections that are achievable.* **CL15:** *They effectively negotiated reasonable adjustments at my initial interview… reduced hours to begin with and reduced days, that was really good … it enabled me to ease into the working full time.* **EM02:** Response when asked about suggested strategies or accommodations: *Visual reminders or breaking down tasks into steps*.

**Table 2.** Quotes from Clients (CL), Family Members (FM), and Employers (EM) that Illustrate Outcomes of Autism EmployABLE (AE)

Category	Subcategory	Example illustrative quotes
Outcomes	**Improved outcomes compared with generic disability employment services**	**CL02:** *My experiences heavily tinged and heavily messed up with many years of … disability employment services just being abusive and not helpful. They’re just there to shove you along… Any sort of pushing I received was pushing into a program or a class or a course or a completely inappropriate thing for me, someone with an accounting degree.* **CL02:** DES staff member told him: *If you just lie on this form about having a bachelor’s degree then the government will pay for it.* **CL03:** *Disability employment services …the way that it’s happening now, it’s a big money grab.* **CL04:** Experience with DES: *They were giving me repetitive jobs that I would get bored of easily. And they were hesitant to give me the bigger jobs.* ***C*L11:** *The program* (AE) *is a lot better than the job agencies I’ve had to use in the past… They’ve actually enabled me to find a job that’s more related to my career. Most of the other* (job agency) *people just wanted to give me the first job they could find.* **FM02:** *I think they’re 100% on the disability employment services…* Experience with DES*: They were basically phone calls that last 10 minutes… And they (AE) are more reliable, actually. Being there for them.* **FM04:** *We’re a little bit angry at the other employment company because it’s like we felt like they didn’t really do anything for* (client) **FM06:** *Son worked for 4 years doing a sort of apprenticeship where he did receive plenty of support. This program targeted people with autism so the support was extensive and well managed.* **EM01:** *The whole experience has been very positive. We had a little bit of exposure to other disability client service providers, and it was far less pleasant than the service team was able to provide.* **EM04:** (*Employer) stated that in comparison to other providers, staff in the EmployABLE program are “… given the time and space to get to know each client, and to understand what they want to do, rather than to push them into work.”*
	**Enhanced independence, happiness**	**Cl06:** *When I work, I feel a sense of independence. It gives me back a sense of purpose in life. Now I can be somebody in the community.* **CL11:** *I’m still living at home at the moment, but … long-term certainty in my contract has enabled me to take further steps towards my independence … I have been looking at houses … to move out.* **CL12:** *I’m very happy with my new job. I’ve been really enjoying it. I’ve met some really nice people. I think I’d love to do it for a long time. I think it has* (made me more independent) *because I catch the bus on my own.* **CL13**: *It’s made me feel very accomplished, it’s like I’ve finally got a job now and I’m getting paid and I’m able to get up every day, …and go do something.* **FM03**: *He’s been very happy to be going in there, and I think he likes that social interaction. His confidence has really grown, he’s working, he feels good about that. He’s saving money and everything’s sort of all coming together for him, which is really good.* **FM04:** *I would say honestly, the anxiety, his OCD, we’ve just noticed a slight increase in that, but we think it’s probably because it’s a new routine for him to get used to. But… the positive things far weigh out that. He’s happier, he’s getting himself to work.* **FM04:** *He’s actually talking to other people as much as talking to family members, he’s out chatting to other people.* **FM06:** *He does look happy but at the same time he can feel the pressure to do the required tasks to keep the job. He seems to be more independent with preparing himself and getting ready to go to work every day.* **EM03:** *He’s really loving the role there. He’s having a laugh with the team, he’s getting his confidence, which is really important*.

**Table 3.** Quotes from Clients (CL), Family Members (FM), and Employers (EM) that Illustrate Satisfaction with Autism EmployABLE (AE)

Category	Subcategory	Example illustrative quotes
Satisfaction with program and suggested improvements	**Happy with support from AE** (no recommended changes, continue program, liked staff)	**CL01:** *They supported me brilliantly…. I can’t really find a flaw.* **CL02:** *I really hope it stays around. If it’s helping me… it must be helping so many other people too.* **CL03:** *I liked the fact that the staff have been really nice, really encouraging, really supportive.* **CL13:** *I don’t know if it needs to be improved, from what my experience with it all, I think it’s been really good.* **FM02:** *I think it’s been all working really, really well. It’s hard to see how it could be made even better… more funding to actually keep going would be a fabulous thing.*
	**More industry-specific knowledge/mentoring**	**CL04:** *In certain niche fields, it can be very difficult to try and find something… I would really like to see an option available for people ... like industry mentors in certain industries who can deal with people who are quite capable, but have issues…* **CL06:** *Having someone who has… more technical knowledge, like in web graphic design, programming, software development so that way you can fine tune the candidates needs for job interviews, cover letters, and also resumes.*
	**More sessions with other participants**	**CL03:** *Sessions with a few other people… What they have personally taken out of the program and whether I could apply what they’ve found and use that for my own purposes.* **CL13:** *Maybe meeting other people… I think people in my family would probably say that’s a good one for me because I don’t get out and try and get to know people much anymore.*
	**More marketing/written materials**	**EM02:** *The only thing really maybe was a couple more fliers. I think workbooks would have been nice.* **EM03:** *If anything, probably more kind of marketing materials or information that can be sent out.*
	**Ability testing**	**EM05:** *Potentially… as part of the program, could do some cognitive and emotional ability, quantitative testing. Just so that when they’re recommending candidates for a field… they know already what their strong and not so strong points are.*

### Category: PEO model

#### Person

Family members commented on the time taken by Autism EmployABLE staff to gain a comprehensive understanding of the clients’ experiences, strengths, and support needs, and to genuinely listen to their employment aspirations (see illustrative quotes in Table 1). Participants spoke about the way that Autism EmployABLE staff helped them to better understand themselves, particularly those who were newly diagnosed and coming to terms with this new identity. This understanding of themselves appeared to help them to share their attributes with employers: *They knew what their strong points are, and where they would feel uncomfortable.* Participants commented on the way that the Autism EmployABLE staff facilitated setting of personally meaningful goals and identifying the steps toward achieving their goals. They also described the focus on the development of foundational skills such as learning how to navigate the social expectations of employment, manage their time, and how to disclose their diagnosis in a positive way. Where needed to meet an employment goal, Autism EmployABLE sourced vocational training externally. Clients and family members commented on the rapport that Autism EmployABLE staff developed with the clients, which enabled them to support their emotional well-being especially those experiencing a loss of confidence or motivation, or mental health challenges. They also observed that the Autism EmployABLE staff aimed to equip the clients with the skills needed to independently make decisions, source jobs, and self-advocate, rather than simply placing them in jobs.

#### Environment

Clients spoke of their fear of negative assumptions about autistic people in the workplace. They welcomed the Autism EmployABLE’s advocacy on their behalf and awareness raising about the value of employing autistic people and adding diversity within workplaces. Employers likewise appreciated the sessions that enhanced their capacity to work with autistic employees. The clients universally described their managers and coworkers as supportive. Conceivably, Autism EmployABLE’s work with employers may have contributed to coworker acceptance of autistic employees, although it cannot be assumed that this was the case. Some of the employers spoke highly of the capacity of Autism EmployABLE staff to recommend candidates who were ideally suited to their businesses.

#### Occupation

An in-depth understanding of the interests and strengths of the clients appeared to assist Autism EmployABLE staff in matching them to appropriate employment opportunities. Clients appreciated Autism EmployABLE’s efforts to find positions that were commensurate with their skills and qualifications, which contributed to job satisfaction. Clients reported that Autism EmployABLE staff supported the logistics of job acquisition, including supporting job seeking, applications, and the interview process through practice interviews and attending the interview to provide feedback. One of the employers commented on *how prepared* (the clients were) *for the interview and for the work role.* Clients, family members, and employers valued the ongoing support of Autism EmployABLE and the fact they did not feel abandoned after the client got a job. In some cases, the Autism EmployABLE staff negotiated reasonable adjustments for the clients, while in other cases, the clients felt sufficiently empowered to self-advocate for their own workplace adjustments. Employers came away from autism awareness sessions with an understanding of adjustments that are commonly effective for autistic employees (e.g., visual cues, breaking tasks down into steps).

### Category: Outcomes of Autism EmployABLE

#### Improved outcomes compared with generic disability employment services

Clients and family members almost universally spoke about feeling disappointed and, in some cases, angry about the services that they had previously received from a DES (see illustrative quotes in Table 2). Many clients complained about being pushed into inappropriate training or jobs. For example, one of the clients had struggled to remain in a busy, customer-facing retail position because of anxiety related to noise sensitivity and social interaction. Others expressed concern about being placed in training or jobs that were not commensurate with their qualifications or skills. One client was especially angry about being told to lie about the fact that he had a degree so that he could be enrolled in a security guard course. Services were often perceived to be cursory and inadequate (e.g., 10-minute phone calls). Some clients perceived DES providers as being incentivized by government funds to place people in inappropriate jobs or training courses (*it’s a big money grab)*. Employers similarly perceived Autism EmployABLE to be more person-centered and pleasant to work with than the services from the DES they had experienced. An exception to these negative experiences was a parent-reported positive previous experience with an autism-specific 4-year apprenticeship.

#### Enhanced independence, happiness

Clients, family members, and employers described increased feelings of confidence, sense of purpose, and happiness that ensued after finding a job. Both clients and family members reported that employment had led to increased independence in areas such as catching public transport and saving to move out of home. Family members and employers noted increased sociability after gaining employment, which in some cases extended beyond the workplace *he’s out chatting to other people*. Nevertheless, two family members observed some increase in anxiety related to *the pressure to do the required tasks to keep the job,* although they perceived the benefits of working to outweigh these concerns.

### Category: Satisfaction with program

All participants expressed satisfaction with the program (see illustrative quotes in Table 1). Many commented on how much they liked the staff. When asked to suggest ways to improve Autism EmployABLE, the majority of clients and family members had no suggestions. They commonly expressed concerns that the program may not continue and strongly recommended that it should be ongoing. Other participants suggested some improvements described below under lessons learned.

### Lessons learned

The Autism EmployABLE team carefully considered the suggested improvements and implemented changes where possible.Two clients recommended further industry-specific support for specialized areas such as IT and engineering. The staff discussed the provision of mentoring by retired industry professionals. Two employers recommended providing further resources such as written and online resources to support staff managing autistic employees. Autism EmployABLE now provides printed materials as part of the autism awareness training sessions for businesses. Some clients suggested social events with other clients. Autism EmployABLE now provides joint support sessions for clients with similar vocational interests. One employer suggested the use of cognitive and emotional ability quantitative testing to fine-tune placement of candidates*.* Autism EmployABLE now uses aptitude testing for specific roles (e.g., online aptitude tests for cyber security roles).

## Discussion

Research question 1 concerned the perceived helpfulness of Autism EmployABLE and ways that it could be improved. The majority of participants described the program as helpful and highly supportive. Some participants were concerned that Autism EmployABLE may not continue and advocated ongoing funding. They suggested a few changes that have been discussed under *Lessons Learned*. Overall, our findings endorse the need for employment services that address the interaction between personal, environmental, and occupational factors to optimize person-to-job fit, thus supporting the PEO model developed by Law et al.^29^ Participants reported that the clients placed by Autism EmployABLE were well-suited to their roles, which contributed to satisfaction with the program. This contrasted with the participants’ experiences with DES, where clients reported being placed into any role to meet the DES performance targets irrespective of their interests, qualifications, or suitability. Six clients who sought support from Autism EmployABLE had a history of achieving little success through a DES program (either no job placements or unsuitable job placements). Consistent with the findings of Hayward et al.,^28^ DES placements were often unsuitable either because they were overqualified or because the environment was inappropriate (e.g., noisy customer-facing environments). In every case, these unsuitable placements were not sustained. Inappropriate placements that are not sustained may result in government investment being wasted. A poor job fit may also erode the person’s self-esteem and motivation to persist with job seeking. Our findings therefore suggest that some DES programs may fail to match the client’s skills and interests (personal factors) with the job tasks (occupational factors) and/or the workplace environment.

In keeping with findings of Hayward et al.,^28^ participants perceived the employment of professional staff with a comprehensive understanding of autism to be critical to the program’s effectiveness. Given that the factors perceived to contribute to the successful employment of autistic people include effective autism awareness programs^18^ and workplace accommodations tailored to autistic employees,^18,20^ it follows that employment programs should be staffed with professionals with a sound understanding of autism. Interestingly, the only other disability employment service that was viewed positively by a participant was an autism-specific program. The employment of staff with experience in the vocational sector further enhanced the team’s efficacy due to their knowledge of employment conditions, training options, and government supports available to jobseekers with disabilities.

The client-led focus of Autism EmployABLE enables clients to exercise choice in the type of jobs for which they apply. Given the significant heterogeneity of the autistic population, employment services should ideally accommodate a range of interests and vocational strengths and support needs (including those with co-occurring conditions such as intellectual disability or specific learning disorders).^26^ The diversity of roles sourced for the clients who participated in this study are a case in point. They comprised low-skilled or semiskilled jobs such as gardening/landscaping, as well as higher skilled jobs such as electrical engineering. The employer awareness training offered by Autism EmployABLE helped to counter stereotypes about the types of roles that suit autistic people.

Clients valued the person-centered, client-led approach that enabled them to set their own employment goals and work with staff to develop personalized “roadmaps” (e.g., *They actually listened to what I actually wanted to do*). Autism EmployABLE staff actively encourage clients to be as independent as possible in seeking and gaining employment and to become effective self-advocates (e.g., *They've given him tools to look for work*). This approach aims to equip clients with the skills needed throughout their working lives, rather than offering a short-term fix by simply placing people in jobs. A conceptual model of self-advocacy proposed by Test et al.^38^ views self-knowledge and the capacity to communicate needs as prerequisites of effective self-advocacy. A sound understanding of self is therefore a critical component of self-advocacy. Recently diagnosed clients particularly appreciated support in understanding and coming to terms with their new diagnosis. Clients learned how to explain their diagnosis to employers in positive ways and to find the right words to effectively articulate their needs. As highlighted by Lindsay et al.,^39^ disclosure of diagnosis in the workplace has benefits when requesting workplace accommodations and in gaining greater acceptance. A survey of 254 autistic adults found that those who disclosed their diagnosis to their employer were more than three times as likely to be employed than those who did not.^40^ Nevertheless, autistic people are understandably wary of the potential risks of disclosure such as stigmatization and discrimination. Autism EmployABLE staff made the clients aware of strategies to mitigate these risks, such as emphasizing their strengths as well as support needs. They enhanced the likelihood of workplace acceptance by explaining the many strengths of autistic employees^12,13^ to employers and by providing on-the-job coaching.

Research question 2 concerned the participants’ perceptions of the clients’ happiness, confidence, and feelings of independence after gaining employment. Many participants observed that the clients showed signs of increased independence, confidence, and sociability. It is possible that these improvements were unrelated to their employment or that they would ensue if they were placed in any job. Nevertheless, as most of the clients who found employment reported that they liked their job and wished to continue in the position, it seems likely that a good person-to-job fit contributed to improved emotional well-being. Consistent with the findings of Baldwin et al.^9^ and Hedley et al.,^16^ participants reported that the experience of being employed provided an enhanced sense of purpose (*I can be somebody in the community*)*.* Some families noticed, however, some increase in anxiety on starting work. Although some anxiety may be expected when adjusting to a new workplace, staff should be aware of the potential need for further on-the-job support to alleviate initial anxiety. Many clients reported that their close relationship with an Autism EmployABLE staff member was helpful in supporting their emotional well-being and mental health *it’s really helped me up to get back on my feet*.

### Limitations and next steps for future research

The qualitative approach provided an in-depth understanding of the participants’ experiences of employment services. Although the perspectives reported here may likely be shared by other autistic people, their family members, and employers, caution must be taken regarding the transferability of findings to different contexts and sociocultural settings.^41^ Given that the program was new with a relatively small number of clients, some demographics were underrepresented (e.g., female clients). Further research is warranted, particularly with respect to the long-term employment outcomes of participants in this type of program. Future studies could compare the long-term outcomes in terms of sustainable employment of (a) programs that focus on short-term “speed-to-placement’ models, and (b) programs that offer nontime-restricted, client-led, autism-specific support.

### Conclusion

These findings underscore the importance of considering the interaction between the person (e.g., vocational interests and strengths), environment (workplace factors), and occupation (e.g., job tasks) when supporting autistic people to find employment.^29^ This study also supports the value of employment programs that are client-led and autism-specific. Notably, this study highlights the need to source employment opportunities to match each client’s unique skills and interests, to achieve the outcome widely recognized as critical to employment success—good person-to-job fit.
