# Supplementary material for: In Search of a Good Person-to-Job Fit: Perspectives of Autistic Clients, Their Family Members, and Employers of a Pilot Autism-Specific Employment Program
Source: Autism Adulthood. 2025 Oct 24;7(5):623–37. doi: 10.1089/aut.2023.0075 (PMC12670677; doi:10.1089/aut.2023.0075)
Supplement: Supplementary file 1 [file aut.2023.0075_supplementaryfile.pdf]

Supplementary file:
Semi-structured interview questions

- **Autism EmployABLE Clients: Semi-structured interview questions**
  - Please tell me about the help you got from Autism EmployABLE staff in finding the job. In what ways has it been helpful or unhelpful in finding a job?

    Probes: If you haven't found a job yet, can you tell me about the ways that the staff have helped you make steps towards getting a job in the future? Did they help you with a resume? Did they help you with interview preparation? Did you have a job trial or work experience?

  - If you have found a job, let's talk about what it is like for you.

    Probes: How happy or unhappy you are with your new job? Is this job something you would like to do for a long time (e.g., a few years), or for a short time before getting a different job? Would you like to move on to other jobs in the future? Would you like to do more study, so that you can get a different job in the future?

  - Please tell me about the help you got from Autism EmployABLE staff in understanding and navigating your workplace.

    Probes: Were the Autism EmployABLE staff helpful or unhelpful working alongside you at your workplace? Did the Autism EmployABLE staff suggest some ways to make your job easier? (for example, providing visual reminders or breaking down tasks into smaller steps). If so, can you tell me how useful these suggestions worked.

  - How supported did you feel by your employer and/or co-workers?

    Probes: Can you tell me some ways you felt supported by them? Can you tell me some ways that you feel you could have been better supported by them?

  - How has working made you feel?

    Probes: Do you think the job has made a difference to how confident you feel about working? How confident you feel as a person? How happy you feel? How independent you feel?

  - Were there some things about the Autism EmployABLE program that you liked?

  - Were there some things about the Autism EmployABLE program that you did not like and that could be improved?

  - Have you ever received support in the past to look for employment, such as from a program at school or through a Disability Employment Service? If yes, how do you think the Autism EmployABLE program compares to these past supports?

- **Family members of Autism EmployABLE clients: Semi-structured interviews questions**
  - What are your thoughts about the job your family member on the autism spectrum has?

    Probes: Is it a good fit for them in the short-term? What about in the long-term?

  - What about the support provided by the Autism EmployABLE staff?

    Probes: Do you think it helped them do the job?

  - What have you noticed about your family member since they have been working?

    Probes: Has there been any effect on their confidence about working? Their confidence generally? How happy they feel? How independent they feel?

  - Can you describe any ways that the service provided by Autism EmployABLE worked well?

  - Can you describe any ways that the service provided by Autism EmployABLE could be improved?

  - Has your family member ever received support in the past to look for employment, such as from a program at school or through a Disability Employment Service? If yes, how do you think the Autism EmployABLE program compares to these past supports?

- **Workplace supervisors/employers of Autism EmployABLE clients: Semi-structured interview questions**
  - Tell me about your experiences of the information and support provided by Autism EmployABLE staff to you and/or your staff.

    Probes: Was it helpful in enabling supervisors and co-workers to understand:

    - autism in general?
    - the way that autism impacts on the individual employee on the spectrum at your workplace?
    - the best way to support the individual worker on the spectrum at your workplace?

    How could this support be improved?

  - Let's talk about the person on the spectrum who you have employed and the support they received from Autism EmployABLE

    Probes: Do you feel that they are a good match for the position?
    What about the job coaching provided to them? Did it help them to do the work successfully?
    Was the frequency and duration of the job coaching sufficient?

Could the job coaching provided to your employee on the spectrum be improved?

- What did you think about the strategies or "accommodations" suggested or provided (e.g., visual reminders, breaking down tasks into steps)?

  Probes: Did they help the employee on the spectrum to do the work successfully? How could they be improved?

- Were there other aspects of the service provided by Autism EmployABLE that worked well?

- Are there ways that the service provided by Autism EmployABLE could be improved.

- Have you previously employed or worked with a person on the autism spectrum?

- Have you previously liaised/collaborated with an autism-specific or disability employment service or program? If yes, how do you think the Autism EmployABLE program compares to other programs or services?
